# Real-time PCR assay for detection of *Staphylococcus aureus*, Panton-Valentine Leucocidin and Methicillin Resistance directly from clinical samples

**DOI:** 10.3934/microbiol.2019.2.138

**Published:** 2019-05-21

**Authors:** Liliana Galia, Marco Ligozzi, Anna Bertoncelli, Annarita Mazzariol

**Affiliations:** Department of Diagnostics and Public Health, University of Verona, Verona, Italy

**Keywords:** *Staphylococcus aureus*, Real-time PCR, Panton Valentine Leucocidin, MRSA, *nuc* gene, coagulase negative staphylococci

## Abstract

Rapid detection of Methicillin Resistant *Staphylococcus aureus* (MRSA) is an important concern for both treatment and implementation of infection control policies. The present study provides an ‘in house’ real-time PCR assay to detect directly *nuc*, *pvl*, and *mecA* genes. The assay is able to perform identification of MRSA, Methicillin-Sensitive *S. aureus*, Methicillin-Resistant Coagulase Negative Staphylococci and the Panton-Valentine leukocidin virulence gene from rectal and pharyngeal swab samples in a screening context. We found an analytical sensitivity of this current Triplex PCR assay of 514 CFU/mL. Analytical specificity was tested with different Gram-positive and Gram-negative species and yielded no false-positive PCR signal. The sensitivity and specificity of the Triplex Real Time PCR were both 100% for these targets when compared with the culture and conventional methods. This assay is readily adaptable for routine use in a microbiology laboratory, as it will enable the implementation of timely and properly guided therapy and infection control strategies.

## 1. Introduction

*Staphylococcus aureus* plays an important role as human pathogen causing a wide range of infections and is a major cause of nosocomial infections [1]. MRSA has emerged as an important public health concern, causing significant morbidity, mortality and prolonged hospitalization and a trend of increasing prevalence in numerous countries and regions. MRSA strains differ from methicillin-sensitive *S. aureus* (MSSA) strains due to the insertion of the staohylococcal cassette chromosome mec (SCCmec) into on the chromosome gene orfX of the staphylococcal cassette chromosome *mec* (SCC*mec*). The SCC*mec* element is a mobile genetic element, which harbors the single determinant for methicillin resistance, namely the *mecA* or *mecC* gene. These genes encode PBP2a enzymes that have low affinity for all β-lactams, except for the fifth generation cephalosporins such as ceftaloridine or ceftobiprole. Acquisition of mecA renders β-lactams useless against MRSA and alternative therapies need to be used in serious infections.

MRSA strains can be distinguished between in hospital-acquired (HA-MRSA) and community-acquired (CA-MRSA). CA-MRSA strains are reported to belong to SCCmec type IV, V, or VI. Compared to HA-MRSA strains, CA-MRSA are usually susceptible to macrolides and fluoroquinolones antibiotics and may produce Panton-Valentine leucocidin (PVL).

PVL, a two component cytotoxin able to form pores that destroy leukocytes [2], accounts also for the high virulence potential of CA-MRSA [3] and may also induce expression of other virulence factors [4]. *S. aureus* strains producing PVL are involved in skin and soft tissue infections (SSTIs), necrotizing pneumonia [5] and osteomyelitis [6]–[8].

Rapid detection of MRSA is imperative for both treatment and implementation of infection control policies to prevent disease spread and outbreaks [9]. Molecular methods, including polymerase chain reaction (PCR) and real-time PCR, have been used for rapid MRSA identification [10]. PVL real-time PCR assay was also developed [11]. Fast and accurate identification of *S. aureus* [12] and detection of methicillin resistance are crucial for immediate treatment with effective antibiotics, which will result in decreased morbidity and mortality rates [13]. Moreover, the PVL locus is a virulence factor, useful for molecular diagnosis [14].

In addition to *S. aureus*, coagulase negative staphylococci (CoNS) have also become increasingly important in recent years. They are particularly associated with the use of indwelling or implanted foreing bodies. Moreover a large proportion of CoNS resulted methicillin resistant (MRCoNS) harbouring the *mecA* gene [15]

The present study describes an ‘in house’ triplex PCR assay developed to identify and differentiate MRSA (Methicillin-Resistant *S. aureus*), MSSA (Methicillin- Sensitive *S. aureus*), MRCoNS (Methicillin-Resistant Coagulase Negative Staphylococci) and to detect the Panton-Valentine leukocidin virulence gene, directly from clinical samples even heavy contaminated as rectal or pharyngeal swabs.

## 2. Materials and methods

A multiplex TaqMan PCR method for the detection of PVL-encoding genes, mecA (for detection of methicillin resistance) and nuc (for identification of S. aureus) genes was initially developed using reference strains, *S. aureus* ATCC 25913 (MSSA) and ATCC 700699 (MRSA).

Both strains were incubated for 24 h on Mannitol Salt Agar (MSA) plates. Nucleic acids were extracted with a Microlab Nimbus apparatus (Hamilton Robotics, NV, USA) from 350 µl of 0.5 McF suspension according to the manufacturer's instructions and recovered in 100 µl of elution buffer.

The nucleotide sequence of *mecA*, *pvl* and *nuc* genes of *S. aureus* were obtained from the GenBank® database and used in the design of primer and probe sequences (Table 1) which were evaluated for specificity using the standard nucleotide comparison tool: BLASTN (www.ncbi.nlm.nih.gov). The probes were labeled with three different fluorescent dyes (FAM, VIC, NED) at the 5′ end and included a minor groove binder molecule and non fluorescent quencer (MGB-NFQ) at the 3′ end.

**Table 1.** primers and probes used for Real-time PCR.

Target	GenBank® Accession number	Primer/Probe	Sequence →	position	Amplicon size
		mecA fw	CAATGCCAAAATCTCAGGTAAAGTG	1931–1957	
*mecA*	KC243783.1	mecA rev	AACCATCGTTACGGATTGCTTC	2018–2018	107
		mecA Probe	FAM-ATGAGCTATATGAGAACGG-MGBNFQ	1958–1979
		pvl fw	AAATGCTGGACAAAACTTCTTGG	606–624	
*pvl*	X72700.1	pvl rev	TTTGCAGCGTTTTGTTTTCG	693–712	108
		pvl Probe	VIC-AAATGCCAGTGTTATCC-MGBNFQ	636–653
		nuc fw	GGCATATGTATGGCAATTGTTTC	25–48	
*nuc*	DQ50738	nuc rev	CGTATTGCCCTTTCGAAACATT	76–97	73
		nuc Probe	NED-ATTACTTATAGGGATGGCTATC-MGBNFQ	49–71

The 25 µl reaction mixture contained 10 µl of DNA template, 2.5 µl of primers (900nM) and probe (250 nM), 12.5 µl 2X Master mix (Applied Biosystem CA, USA) Multiplex real-time PCR was performed on an ABI 7500 real-time PCR system (Applied Biosystems).

**Figure 1.**
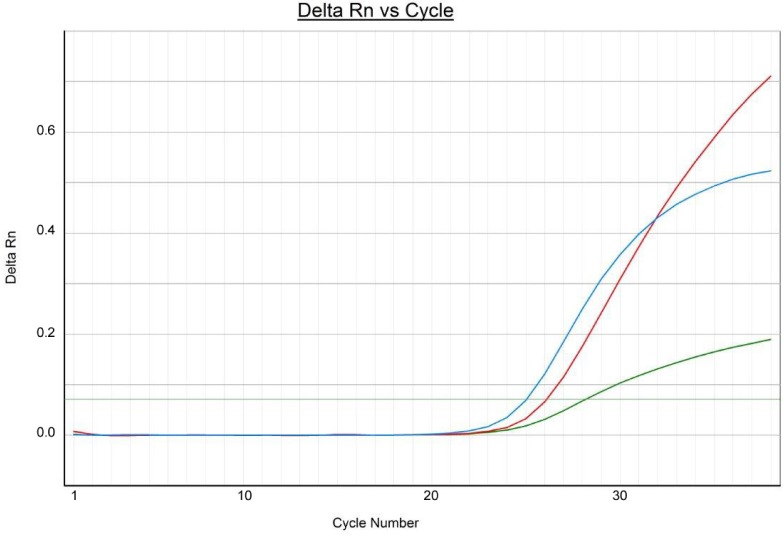
Amplification plot obtained with a detection system based on the use of three fluorophores with strain *S. aureus* MDR1305: FAM (red line) for the detection of *mecA* gene, VIC (blue line) for the detection of *pvl* gene and NED (green line) for the detection of *nuc* gene.

Thermocycler conditions consisted of an initial denaturation at 94 °C for 5 min, followed by amplification that was performed during 38 cycles of denaturation at 95 °C for 15 sec and annealing/extension at 60 °C for 30 sec. Multiple fluorescent signals were obtained once per cycle upon completion of extension step. Data acquisition and analysis of the real-time PCR assay were performed using ABI 7500 real-time PCR system (Applied Biosystems). Figure 1 shows an example of the amplification plot of the three genes using the *S. aureus* MDR1305 as representative strain.

The Real-Time protocol was confirmed with 40 *S. aureus* strains, either MRSA (25) and MSSA (15) and 4 *S. aureus* strains producing PVL (including in MRSA group), identify by conventional culture method and by endpoint PCR for methicillin resistance and *pvl* gene. The specificity was checked using other *Staphylococcus* spp strains, other Gram-positive and Gram-negative. All *S. aureus* strains were recognized by amplification of the *nuc* gene, while there was no amplification of this gene by other *Staphylococcus* spp, or other bacteria species.

**Figure 2.**
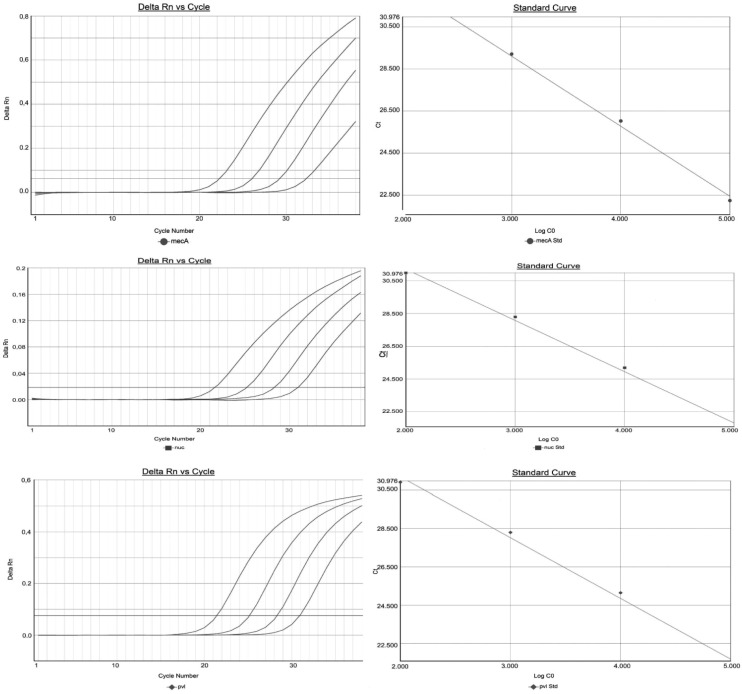
Amplification curves and dilution end point standard curves of log10 genome versus Ct cycle number of nuc (green), mecA (red) and pvl (blue) genes.

The analytical sensitivity was evaluated using serial dilutions of cell stocks. The sensitivity of the assay was evaluated using 2 MRSA clinical isolates, 2 MSSA clinical isolates, and the reference strains ATCC 25923 (MSSA) and ATCC 700699 (MRSA). A serial dilution of cultured *S. aureus* ranging from 5 × 10^8^ to 5 × 10 CFU/ml n NaCl was analyzed. To establish the Limit of Detection (LoD), genomic DNA extracted from each dilution was tested in 5 replicates. The lowest concentration of serial dilution that yielded positive test results in 95% he replicates were set as the LoD (approximately 18 copies/reaction). The analytical sensitivity curve was constructed by plotting the *S. aureus* DNA reference strains input against the corresponding PCR threshold cycle (*C_t_*) values. Our assay showed an efficiency > 90% with a correlation coefficient R^2^ > 0.99. The linearity of this assay is detectable in 10-fold scalar dilutions (n = 5 for each dilution) from 5 × 10^6^ copies/mL to 5 × 10^2^ copies/mL. To evaluate intra-assay and inter-assay reproducibility, we calculated the coefficient of variation (CV) of the threshold cycle (*CT*) values of serial DNA specific for *mecA*, *nucA* and *pvl* target gene in triplicate. We assayed intra-assay analysis with 10-fold serial dilutions (from 5 × 10^5^ copies/mL to 5 × 10^2^ copies/mL) of the same target DNA. The analysis of coefficient of variation (CV) calculated on Ct average was less than 2% for all dilutions of *mecA*, *nucA* and *pvl* DNA target. Figure 2 shows the amplification curves and dilution end point standard curves of log_10_ genome versus Ct cycle number of *nuc* (green), *mecA* (red) and *pvl* (blue) genes. The analytic sensitivity of this assay for each gene was approximately 500 CFU/mL.

Analytical specificity was evaluated using DNA lysates prepared from 20 phenotypically and genotypically well-characterized methicillin sensitive *Staphylococcus* *spp*., namely *S. haemolyticus* (8), *S. hominis* (4), *S. epidermidis* (8), and 13 other Gram-positive, namely *Enterococcus faecalis* (9), *Enterococcus faecium* (4), and some Gram-negative strains usually found in rectal swabs such as *Escherichia coli* (20), *Proteus vulgaris* (8), *Enterobacter cloacae* (8), *Klebsiella pneumoniae* (10).

Routinely, we screen pharyngeal and rectal swabs, collected using eSwab (Copan, Brescia, Italy), for MRSA during surveillance of multi-drug-resistant program. Screening is conducted streaking samples on Mannitol Salt Agar plus a cefoxitin disk (30 µg) and Columbia blood agar homemade plates with 5% sheep blood. Positive strains were grown for 24 h and identified by MALDI-TOF (Bio-Mérieux). The methicillin resistance was tested by Vitek2 (BioMérieux) following EUCAST interpretation guidelines.

A retrospective assay was conducted on 38 pharyngeal and 42 rectal swabs collected from 80 hospitalized patients. All samples were analyzed in parallel using both culture-based method and PCR-based analysis (Table S1). Viable bacterial counts were determined after 18 h of growth at 37 °C. All isolates were identified by MALDI-TOF procedure and, subsequently, tested against antimicrobial agents through broth micro-dilution procedure and EUCAST interpretation criteria (http://www.eucast.breakpoints.org). *S. aureus ATCC 25923* and *S. aureus* ATCC 700699 were used as quality control. Methicillin resistant *S. aureus* isolates carrying the *mecA* gene were evaluated by cefoxitin disk diffusion test (30 µg). According to the Eucast guidelines, a zone of growth inhibition around the cefoxitin disk of < 22 mm indicates the presence of *mecA* gene and the isolate should be reported as MRSA. All isolated *S. aureus* strains were also checked for *mecA* gene by end-point PCR [16].

## 3. Results and discussion

The *pvl* were amplified only by the four *S. aureus* know to harbor this gene. In the same way *mecA* gene was detected only in the 25 MRSA strains and in the MRCoNS.

We found an analytical sensitivity of this current Triplex PCR assay of 514 CFU/mL. PCR assays, with three replicates per sample, consistently detected MRSA alone at 18 copies per reaction.

The analytical specificity evaluated with different *Staphylococcus* spp, Gram positive and Gram-negative strains was of 100% since none of the strains used to test specificity amplified *nuc, pvl and mecA* genes and no false positive was registered.

**Figure 3.**
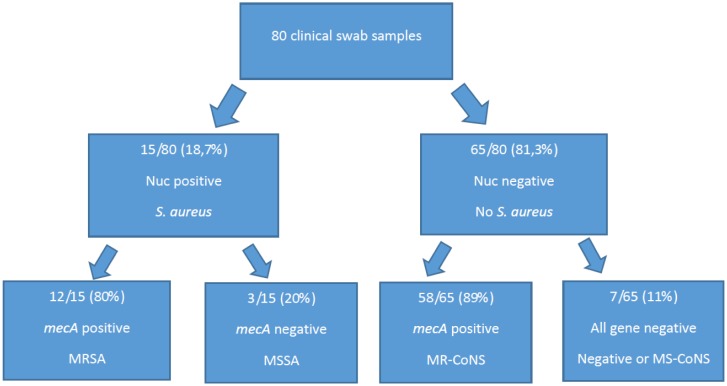
Results summary of Triplex real time PCR assay applied directly to 80 clinical samples.

We applied our triplex RT-PCR assay on 80 clinical samples analyzed in parallel with culture method and MALDI-TOF identification, as summarized in Figure 3. No positive samples were detected for *pvl* gene. The agreement between culture method/MALDI-TOF procedure and our molecular assay was of 100%. The sensitivity and specificity of the Triplex real time RT-PCR were both 100% for these targets when compared with the culture and conventional methods.

This triplex real time PCR assay is able to simultaneously identify the species, the meticillin resistance and the presence of the necrotizing toxin present in the community strains in only one hour, directly from the clinical sample, thus reducing reporting times and activating immediately clinical therapy or decolonization measures

The results obtained with the triplex real-time PCR assay were compared with the results from a culture method, considered as the standard method. This assay was able to differentiate between *S. aureus* and CoNS. In addition, the triplex real time PCR assay differentiates methicillin-susceptible and methicillin-resistant strains and the presence of PVL toxin gene locus, and detecting *nuc*, *pvl*, and *mecA* genes is able to perform identification of MRSA, MSSA, PVL-positive MRSA (PPMRSA), PVL-positive MSSA (PPMSSA), methicillin resistant CoNS (MRCoNS). No unrelated Gram-positive and Gram-negative species yielded any false-positive PCR signal. This assay is readily adaptable for use in routine microbiology laboratory, as it will enable the implementation of timely and properly guided therapy and infection control strategies. The assay provides a valuable tool for the rapid and accurate characterization of staphylococci, as automatic DNA extraction directly from sample, amplification cycles and post-PCR analyses are completed in less than 3 hours. Comparing to the assay developed by Söderquist et al. [17], our assay doesn't need to pre-treat clinical samples by broth enriched method. Our assay was validate starting directly from heavy contaminated clinical samples (rectal swab and pharingeal swab) during multidrug resistant screening program and show a very high sensititity and specificity. Okolie et al. [18] developed a pentaplex PCR assay, using the *spa* gene marker indeed of *nuc* gene for *S. aureus* detection, that is usually used. Added markers were the bacterial marker 16S rRNA and a marker for coagulase-negative staphylococci. The last will be a good improvement of our assay. Okolie et al. [18] tested their assay in spiked bloodcultures which generally have a higher bacterial load than clinical samples.

In conclusion, we develop a highly specific and sensitive triplex real-time PCR assay to apply directly from clinical swab specimens to detect MRSA, MSSA both in combination with *pvl* gene and MRCoNS without any need of broth enrichment step. This assay might be useful in the microbiological screening of colonized patients, especially those who need to undergo heart surgery and need to be decolonized for staphylococci.
