# Supplementary material for: Real-time PCR assay for detection of Staphylococcus aureus, Panton-Valentine Leucocidin and Methicillin Resistance directly from clinical samples
Source: AIMS Microbiol. 2019 May 21;5(2):138–46. doi: 10.3934/microbiol.2019.2.138 (PMC6642910; doi:10.3934/microbiol.2019.2.138)
Supplement: Supplementary file 1 [file microbiol-05-02-138-s001.pdf]

Table S1: results of standard culture method and Real-Time PCR assay directly from 80 clinical specimens.

Samples	Culture Identification		Triplex Real Time PCR assay			Interpretation
	Specimen	ID MALDI	*nuc*	*mecA*	*pvl*	
1	Rectal swab	*S. haemoliticus*	-	+	-	MRCoNS
2	Rectal swab	*S. haemoliticus*	-	+	-	MRCoNS
3	Rectal swab	*S. haemoliticus*	-	+	-	MRCoNS
4	Pharyngeal swab	*S aureus*	+	+	-	MRSA
5	Rectal swab	*S cohnii*	-	-	-	Neg
6	Pharyngeal swab	*S aureus*	+	+	-	MRSA
7	Rectal swab	*S. haemoliticus*	-	+	-	MRCoNS
8	Rectal swab	*S aureus*	+	+	-	MRSA
9	Pharyngeal swab	*S aureus*	+	+	-	MRSA
10	Rectal swab	*S. haemoliticus*	-	+	-	MRCoNS
11	Pharyngeal swab	*S. haemoliticus*	-	+	-	MRCoNS
12	Rectal swab	*S. haemoliticus*	-	+	-	MRCoNS
13	Pharyngeal swab	*S. haemoliticus*	-	+	-	MRCoNS
14	Pharyngeal swab	*S. haemoliticus*	-	+	-	MRCoNS
15	Pharyngeal swab	*S epidermidis*	-	-	-	Neg
16	Pharyngeal swab	*S aureus*	+	+	-	MRSA
17	Rectal swab	*S. haemoliticus*	-	+	-	MRCoNS
18	Rectal swab	*S. haemoliticus*	-	-	-	Neg
19	Rectal swab	*S. haemoliticus*	-	+	-	MRCoNS
20	Pharyngeal swab	*S. haemoliticus*	-	+	-	MRCoNS
21	Rectal swab	*S. haemoliticus*	-	-	-	Neg
22	Rectal swab	*S. haemoliticus*	-	+	-	MRCoNS
23	Pharyngeal swab	*S aureus*	+	-	-	MSSA
24	Pharyngeal swab	*S aureus*	+	-	-	MSSA
25	Pharyngeal swab	*S. haemoliticus*	-	+	-	MRCoNS
26	Rectal swab	*S. haemoliticus*	-	+	-	MRCoNS

27	Rectal swab	*S. haemoliticus*	-	+	-	MRCoNS
28	Pharyngeal swab	*S. haemoliticus*	-	+	-	MRCoNS
29	Rectal swab	*S. haemoliticus*	-	+	-	MRCoNS
30	Pharyngeal swab	*S. haemoliticus*	-	+	-	MRCoNS
31	Rectal swab	*S. haemoliticus*	-	+	-	MRCoNS
32	Pharyngeal swab	*S. haemoliticus*	-	+	-	MRCoNS
33	Rectal swab	*S aureus*	+	+	-	MRSA
34	Rectal swab	*S. haemoliticus*	-	+	-	MRCoNS
35	Rectal swab	*S. haemoliticus*	-	+	-	MRCoNS
36	Rectal swab	*S. haemoliticus*	-	+	-	MRCoNS
37	Pharyngeal swab	*S. haemoliticus*	-	+	-	MRCoNS
38	Pharyngeal swab	*S aureus*	+	+	-	MRSA
39	Rectal swab	*S. haemoliticus*	-	+	-	MRCoNS
40	Pharyngeal swab	*S. haemoliticus*	-	+	-	MRCoNS
41	Pharyngeal swab	*S. haemoliticus*	-	+	-	MRCoNS
42	Rectal swab	*S. haemoliticus*	-	+	-	MRCoNS
43	Pharyngeal swab	*S. haemoliticus*	-	-	-	Neg
44	Rectal swab	*S. haemoliticus*	-	+	-	MRCoNS
45	Rectal swab	*S. haemoliticus*	-	+	-	MRCoNS
46	Pharyngeal swab	*S aureus*	+	+	-	MRSA
47	Rectal swab	*S. haemoliticus*	-	+	-	MRCoNS
48	Pharyngeal swab	*S. haemoliticus*	-	+	-	MRCoNS
49	Rectal swab	*S. haemoliticus*	-	-	-	Neg
50	Rectal swab	*S. haemoliticus*	-	+	-	MRCoNS
51	Pharyngeal swab	*S. haemoliticus*		+	-	MRCoNS
52	Rectal swab	*S. haemoliticus*	+	+	-	MRSA
53	Pharyngeal swab	*S. haemoliticus*	-	+	-	MRCoNS
54	Rectal swab	*S. haemoliticus*	-	+	-	MRCoNS
55	Rectal swab	*S. haemoliticus*	-	+	-	MRCoNS
56	Pharyngeal swab	*S. haemoliticus*	+	+	-	MRSA
57	Rectal swab	*S. haemoliticus*	-	+	-	MRCoNS
58	Pharyngeal swab	*S. haemoliticus*	-	+	-	MRCoNS

59	Pharyngeal swab	*S. haemoliticus*	-	+	-	MRCoNS
60	Rectal swab	*S. haemoliticus*	-	+	-	MRCoNS
61	Rectal swab	*S. haemoliticus*	-	+	-	MRCoNS
62	Pharyngeal swab	*S. haemoliticus*	-	+	-	MRCoNS
63	Pharyngeal swab	*S. haemoliticus*	-	+	-	MRCoNS
64	Pharyngeal swab	*S. haemoliticus*	-	+	-	MRCoNS
65	Rectal swab	*S. haemoliticus*	-	+	-	MRCoNS
66	Rectal swab	*S. haemoliticus*	-	+	-	MRCoNS
67	Pharyngeal swab	*S aureus*	+	+	-	MRSA
68	Rectal swab	*S. haemoliticus*	-	+	-	MRCoNS
69	Rectal swab	*S. haemoliticus*	-	+	-	MRCoNS
70	Pharyngeal swab	*S aureus*	+	+	-	MRSA
71	Pharyngeal swab	*S aureus*	+	-	-	MSSA
72	Pharyngeal swab	*S. haemoliticus*	-	+	-	MRCoNS
73	Rectal swab	*S. haemoliticus*	-	+	-	MRCoNS
74	Rectal swab	*S. haemoliticus*	-	+	-	MRCoNS
75	Pharyngeal swab	*S. haemoliticus*	-	+	-	MRCoNS
76	Pharyngeal swab	*S. haemoliticus*	-	+	-	MRCoNS
77	Rectal swab	*S. cohonii*	-	-	-	Neg
78	Pharyngeal swab	*S. haemoliticus*	-	+	-	MRCoNS
79	Rectal swab	*S. haemoliticus*	-	+	-	MRCoNS
80	Pharyngeal swab	*S. haemoliticus*	-	+	-	MRCoNS
ATCC 700699			+	+	-	
ATCC 25923			+	-	+	
